# Molecular classification of breast cancer using the mRNA expression profiles of immune-related genes

**DOI:** 10.1038/s41598-020-61710-y

**Published:** 2020-03-16

**Authors:** Juan Mei, Ji Zhao, Yi Fu

**Affiliations:** School of Internet of Things Engineering, Wuxi City College of Vocational Technology, Wuxi, 214153 China

**Keywords:** Data mining, Functional clustering, Machine learning

## Abstract

Breast cancer is the most lethal cancer in women and displaying a broad range of heterogeneity in terms of clinical, molecular behavior and response to therapy. Increasing evidence demonstrated that immune-related genes were an important source of prognostic information for several types of tumors. In this study, the k-mean clustering was applied to gene expression data from the immune-related genes, two molecular clusters were identified for 1980 breast cancer patients. The prognostic significance of the immune-related genes based classification was confirmed in the log-rank test. These clusters were also associated with immune checkpoints, immune-related features and tumor infiltrating levels. In addition, we used the shrunken centroid algorithm to predict the cluster of a given breast cancer sample, and good predictive results were obtained by this algorithm. These results indicated that the proposed classification method is a promising method, and we hope that this method may improve the treatment stratification of breast cancer in the future.

## Introduction

Breast cancer is one of the most aggressive cancers with an estimated 2100000 new cases and 627000 deaths worldwide in 2018^1^. During the past years, multidisciplinary treatment regimen, such as surgery, chemotherapy, radiotherapy, hormonal therapy and targeted therapy had been made much progress for breast cancer^2,3^. The five-year survival rate of breast cancer was approximately 85% and even worse for breast cancer patients with advanced stage. In recent years, the gene expression profiles in breast cancer patients had been investigated by many studies, and found that this cancer was composed of distinct molecular subtypes^2,4–7^. These distinct molecular subtypes may underlie the high variability of clinical outcomes in breast cancer patients. Therefore, breast cancer should not be considered as a homogeneous entity, and molecular classification of breast cancer into clinically and biologically meaningful subtypes was needed.

At present, several researches had shown that the immune system was one of the determining factors during tumor initiation and progression^8–11^. Several studies illustrated that the presence of tumor infiltrating lymphocytes was often associated with better prognosis several cancer types, including breast cancer^12–21^. Thus, inclusion of immune signatures in the molecular subtyping may provide additional information beyond routine prognosis in breast cancer. However, until now, no attempt has been made to use these immune signatures to stratify breast cancer.

In this study, by using the gene expression profiles of immune-related genes with favorable prognosis, the k-means clustering was applied on the breast cancer samples to establish a robust molecular classification. Then, the associations between the molecular clusters and prognosis, clinicopathological factors, immune-related features and tumor infiltrating levels were assessed. The shrunken centroid algorithm was used to classify the clusters by the gene expression profiles of immune-related genes with favorable prognosis as the input parameters, and good predictive results were obtained in this study.

## Results

### Immune landscape of 17 immune cell types

**Figure 1:**
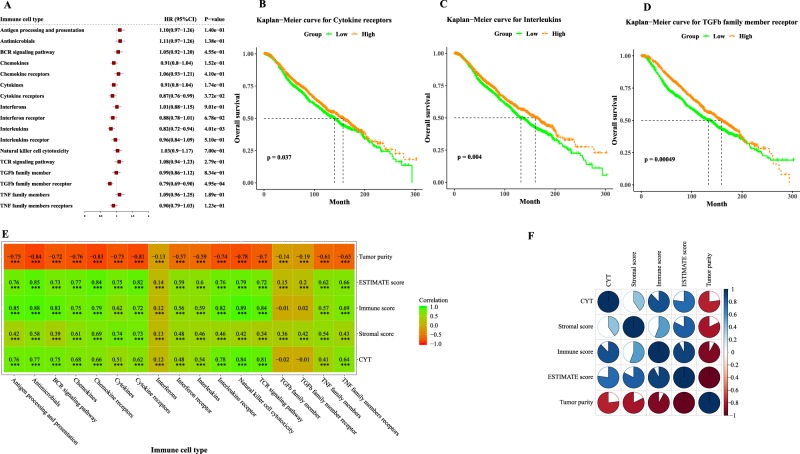
The immune landscape of 17 immune cell types. (**A**) Forest plot visualizing hazard ratios (HRs) with 95% CI and P-values for 17 immune cell types. Kaplan-Meier survival curves by high and low tumor infiltrating levels for **(B)** cytokine receptors, **(C)** interleukins and **(D)** TGFb family member receptor. **(E)** The spearman’s correlation between the tumor infiltrating levels of 17 immune cell types and tumor purity, ESTIMATE score, stromal score, immune score and CYT. Statistical significance at the level of null ≥0.05, * < 0.05, ** < 0.01 and *** < 0.001. **(F)** The Spearman’s correlation between tumor purity, ESTIMATE score, stromal score, immune score and CYT.

We first examined whether the tumor infiltrating levels of 17 immune cell types were the prognostic factors in overall survival of breast cancer. The breast cancer patients were classified into two equal groups by using the median the ssGSEA score as the cutoff point. The univariate Cox analysis indicated that the higher tumor infiltrating levels of cytokine receptors, interleukins, and TGFb family member receptor were significantly associated with favorable prognosis in breast cancer patients (Fig. 1A–D). For example, patients with the high tumor infiltrating level of cytokine receptors had about 0.13 reduced risk of death compared with patients with the low tumor infiltrating level of cytokine receptors.

Using the ESTIMATE algorithm, the tumor purity, ESTIMATE score, immune score and stromal score were estimated, and the Spearman’s correlations between them with the tumor infiltrating levels of 17 immune cell types were calculated (Fig. 1E). The tumor purity, ESTIMATE score, immune score and stromal score were weakly to moderately correlated with the tumor infiltrating levels of 17 immune cell types. In addition, most of the 17 immune cell types shown significant correlations with CYT. The significant associations between tumor purity, ESTIMATE score, immune score, stromal score and CYT were also illustrated in Fig. 1F.

### The cluster analysis of breast cancer

**Figure 2:**
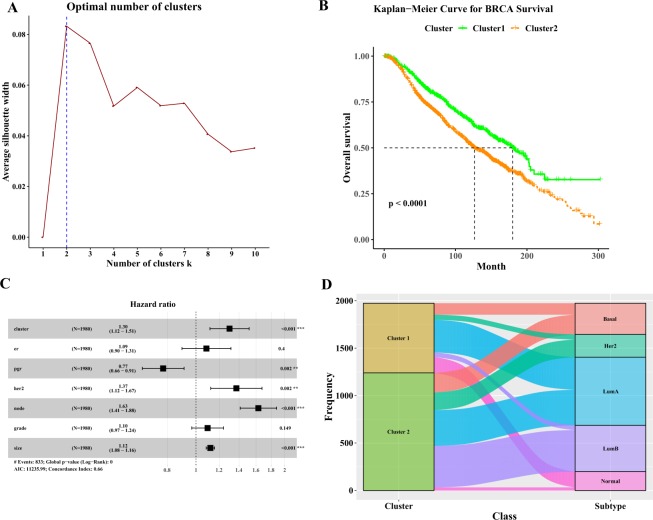
The cluster analysis of breast cancer. (**A**) The silhouette width analysis of the clustering by using the expression profiles of 117 immune-related genes. **(B)** Kaplan-Meier estimate of the overall survival for cluster 1 and cluster 2 patients. **(C)** The multivariable cox regression analyses in breast cancer patients. **(D)** Alluvial diagram for cluster 1 and cluster 2 versus different subtypes in in breast cancer patients.

Then the univariate Cox regression analysis was conducted for assessing the correlation between the expression levels of 2498 immune-related genes and overall survival in the breast cancer cohort. 117 immune-related genes were considered to be correlated with overall survival of breast cancer with the criterion of P-value < 0.05 and hazard ratio (HR) < 1. These survival-related genes were selected for further cluster analysis. The k-means clustering was applied to cluster breast cancer samples based on the expression levels of 117 immune-related genes, and Nbclust testing was applied to determine the optimal number of stable clusters. According to the average silhouette width from the k-means clustering, 2 clusters were chosen as the optimal number of clusters (Fig. 2A). Patients in cluster 1 had significantly longer median overall survival than those in the cluster 2 (180 months versus 127 months; log-rank test P-value < 0.0001) (Fig. 2B). The multivariate Cox regression analysis revealed that the 117 immune-related genes derived clusters, together with progesterone receptor (PGR), HER2, node and size remained an independent prognostic factor (Fig. 2C). We then investigated the distribution of intrinsic molecular subtypes within the clusters. An imbalance in term of intrinsic molecular subtype was noticed (Fig. 2D). Her2 tumors and Luminal B tumors were more likely to be enriched in cluster 2, and Normal like tumors were more likely to be enriched in cluster 1.

### Characterization of immune infiltration profiles between two clusters

**Figure 3:**
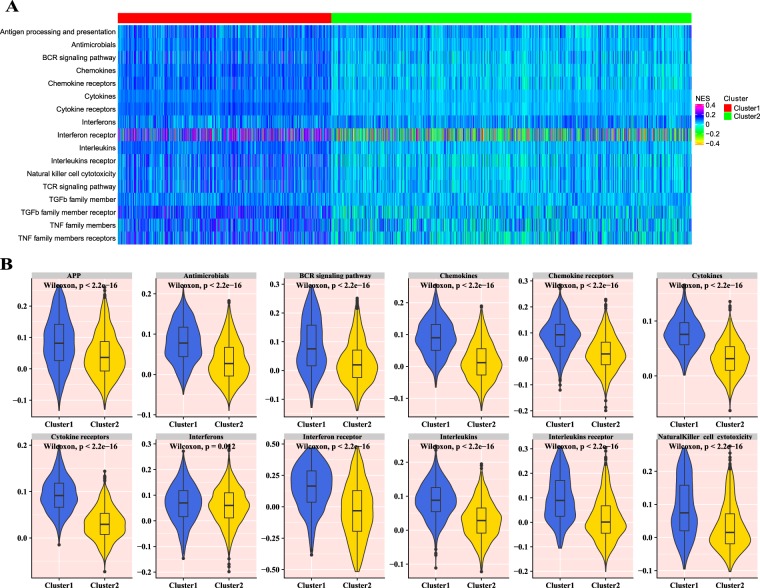
The immune infiltrate profile of two clusters. (**A**) The heatmap of the ssGSEA scores for 17 immune cell types in cluster 1 and cluster 2. **(B)** The violin plots of the tumor infiltrating levels in 12 immune cell types for in cluster 1 and cluster 2. APP: antigen processing and presentation.

The relative tumor infiltrating levels of 1980 breast cancer samples were quantified by using mRNA expression data of 2498 immune-related genes related to 17 immune cell types obtained from the ImmPort database (Fig. 3A). As illustrated in Fig. 3A, the cluster 1 samples were marked by the high level of immune infiltration level, whereas by contrast, the cluster 2 samples were characterized by the low immune infiltration level. These results revealed a comprehensive picture of tumor infiltrating levels, the heterogeneity across samples and differences in immune cell types. Then, the differences in the tumor infiltrating levels of 17 immune cell types between two clusters were also investigated. According to the Wilcoxon test, there were statistically significant differences in these immune cell types between the two clusters in the breast cancer patients (Figs. 3B and S1). The mean tumor infiltrating levels of the cluster 1 were significantly higher than those of the cluster 2.

**Figure 4:**
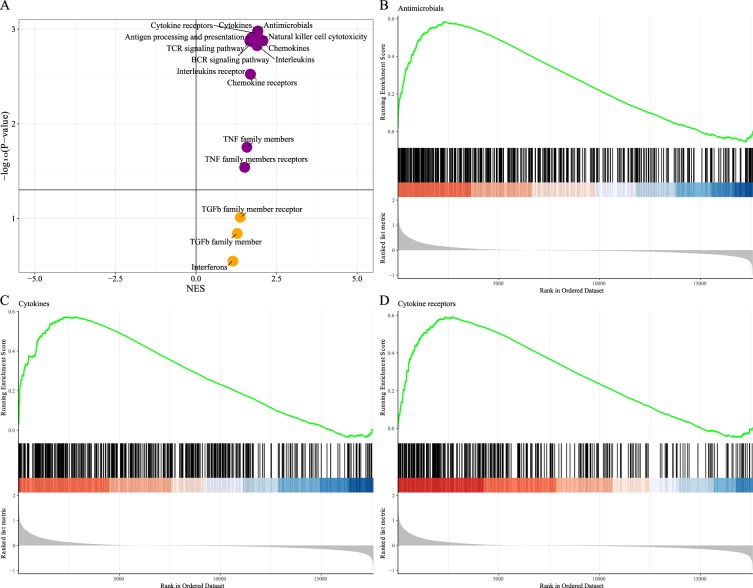
The GSEA delineated the enriched immune cell types. (**A**) Volcano plot of GSEA for the cluster 1 when compared with the cluster 2. GSEA of **(B)** antimicrobials, **(C)** cytokines and **(D)** cytokine receptors for the cluster 1 when compared with the cluster 2.

We then compared the gene expression profile of cluster 1 samples with cluster 2 samples by the GSEA algorithm to determine how the tumor infiltrating levels differed between two clusters. 2498 immune-related genes of 17 immune cell types were selected as the reference gene set. The GSEA for the enriched and depleted immune cell types were illustrated in Figs. 4A–D and S2. Compared with the cluster 2 samples, the cluster 1 samples were significantly enriched with antimicrobials, cytokines, cytokine receptors, antigen processing and presentation, natural killer cell cytotoxicity, chemokines, TCR signaling pathway, BCR signaling pathway, interleukins, interleukins receptor, chemokine receptors, TNF family members and TNF family members receptors.

**Figure 5:**
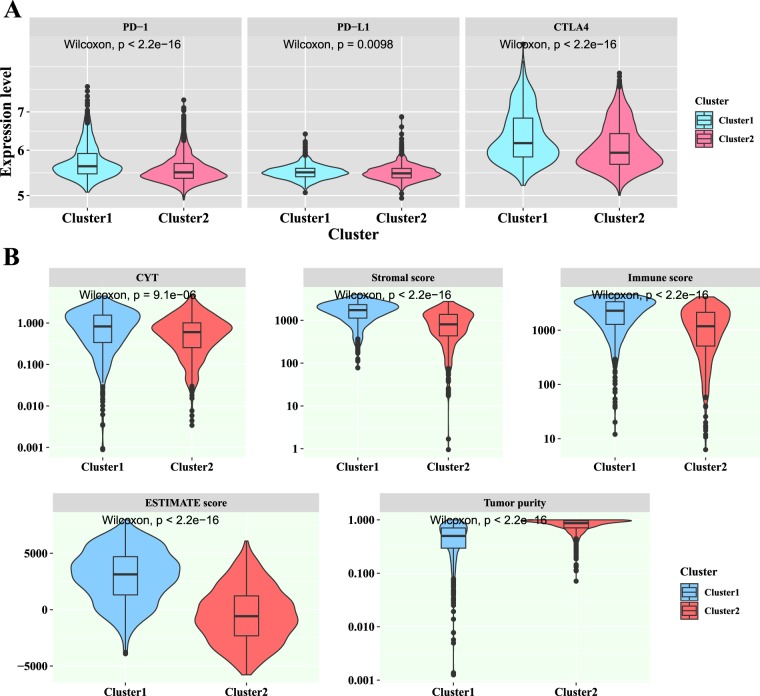
The violin plots of three immune checkpoints and five immune related indices between two clusters. (**A**) The violin plots of the PD-1, PD-L1 and CTLA4 expression levels for two clusters. **(B)** The violin plots of the CYT, stromal score, immune score, ESTIMATE score and tumor purity for two clusters.

Immune checkpoints are critical modulators in the immune system, allowing the initiation of a productive immune response and preventing the onset of autoimmunity. Among these immune checkpoints, PD-1, PD-L1 and CTLA-4 were the most important immune checkpoints. In this study, we wanted to know whether the expression levels of PD-1, PD-L1 and CTLA4 were different between two breast cancer clusters. For doing this, the Wilcoxon test was applied to calculate the difference between the two breast cancer clusters in the expression levels of PD-1, PD-L1 and CTLA4. As illustrated in Fig. 5A, the expression levels of PD-1, PD-L1 and CTLA4 of the cluster 1 were significantly higher than those of the cluster 2.

The differences between the two breast cancer clusters in CYT, immune score, ESTIMATE score, stromal score and tumor purity were also investigated in the breast cancer patients (Fig. 5B). Among these four indices, the CYT, calculated from the geometric mean of the expression of the genes GZMA and PRF1, was used to reflect the patient’s antitumoral immune cytolytic activity and the immune score was used to reflect the infiltration of leukocytes. As illustrated in Fig. 5B, the average values of the CYT, immune score, ESTIMATE score and stromal score in the cluster 1 were significantly higher than those in the cluster 2. These results were expected, as the tumor infiltrating levels of 17 immune cell types in the cluster 1 were higher than those in the cluster 2, and the CYT, immune score, ESTIMATE score and stromal score were significantly correlated with most of immune cell types (all P-values < 2.20E-16; Wilcoxon test). Tumor purity is defined as the proportion of cancer cells in the tumor tissue^22^. The average tumor purity of the cluster 2 was significantly higher than that of the cluster 1 (P-value < 2.20E-16; Wilcoxon test). This result was expected, as previous published works suggested that the immune cells were negatively correlated with tumor purity at the pan-cancer level^22^.

### Generation of the breast cancer classifier

**Figure 6:**
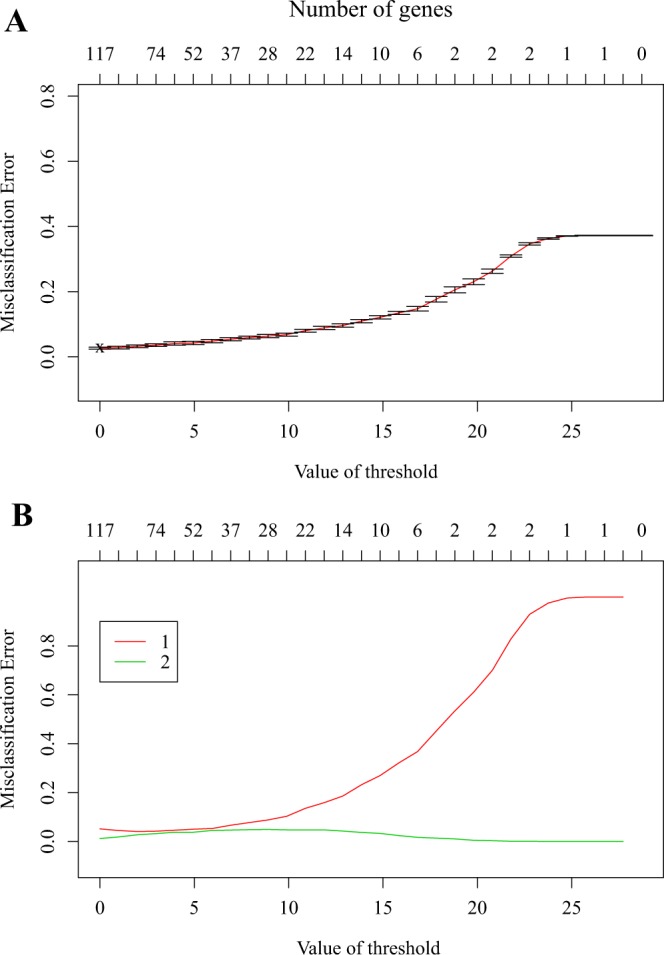
The predictive results for two clusters by the shrunken centroid algorithm. (**A**) The misclassification errors for predicting the two clusters with different parameters. **(B)** The misclassification errors for predicting the cluster 1 and cluster 2 with different parameters.

In this study, we wanted to build a classifier that could identify the cluster of the breast cancer patients by using the expression profile of 117 immune-related genes. For doing this, the shrunken centroid algorithm^23^ that implemented in the R package pamr (version 1.55) was used to learn a classifier for discriminating between cluster 1 and cluster 2. The ten-fold cross-validation was performed to select the optimal threshold for centroid shrinkage. The shrunken centroid algorithm identified a set of 117 signature genes with the most robust model that minimized the overall misclassification error. These 117 signature genes were used to predict the cluster of the breast cancer samples with the misclassification rate of 2.68% of the two tumor clusters (Fig. 6A). The predictive ability of the shrunken centroid algorithm for prediction each cluster was illustrated in Fig. 6B. These predictive results clearly indicated that the shrunken centroid algorithm was suitable to prediction the cluster of breast cancer samples (Fig. 6B).

## Discussion

The breast cancer patients often display a heterogeneous clinical outcome. Given the heterogeneity of breast cancer patients, it is important to determine the appropriate treatment for patients diagnosed with breast cancer. Therefore, understanding the heterogeneity of breast cancer is one of the most fundamental goals in breast cancer. In the past few years, using mRNA expression profiles to stratify tumors into different molecular subtypes have been applied in several types of tumors^24–28^. Here, in this study, by using the mRNA expression profile of 117 immune-related genes with favorable prognostic factor, the k-means clustering was applied to cluster the breast cancer patients without applying any clinical or biological information. By analyzing the associations between the clusters and clinical outcome of breast cancer patients, we found that the 117 immune-related genes derived clusters were significantly associated with the overall survival, and the clusters was an independent prognostic factor in the multivariate Cox proportional-hazard analysis.

Compared with patients in cluster 2, patients in cluster 1 had higher tumor infiltrating levels, CYT, immune score and stromal score. The expression levels of PD-1, PD-L1 and CTLA4 in the cluster 1 were also significantly higher than those in cluster 2. Benefit from the meaningful results of clustering breast cancer patients, we will strive to use the mRNA expression profile of 117 immune-related genes in other tumors to classify patients into distinct clusters in our future work. To this end, a computational model was built to predict the molecular clusters defined by the expression levels of the immune-related genes.

The naïve bayes, logistic, IBK, J48, random forest and libSVM that implemented in Weka (version 3.8.2)^29^ were applied to compare the predictive results with the shrunken centroid classifier, the default parameters of these algorithms in Weka were used. The expression profile of 117 immune-related genes was used as the parameters of these classifiers. The ten-fold cross-validation was used to evaluate the performance of these classifiers. The overall accuracies of the naïve bayes, logistic, IBK, J48, random forest, libSVM and shrunken centroid were 95.05%, 97.12%, 89.39%, 88.79%, 95.61%, 96.57% and 97.32%, respectively (Table 1). These predictive results clearly demonstrated that the overall accuracy of the shrunken centroid classifier was higher than those of other classifiers, and the shrunken centroid classifier was a promising algorithm in prediction of the clusters of breast cancer patients.

**Table 1:** The predictive results of different input parameters.

Algorithms	117 features	50 features	84 features	112 features
Naïve bayes	95.05%	94.75%	96.01%	94.85%
Logistic	97.12%	96.41%	96.77%	95.40%
IBK	89.39%	89.55%	91.06%	89.49%
J48	88.79%	89.55%	89.09%	88.43%
Random forest	95.61%	94.80%	94.64%	95.45%
libSVM	96.57%	96.57%	96.82%	96.87%

(In this table, the 50 features were selected by the mRMR, the 84 features with the P-values less than 0.001 were selected by ANOVA and the 112 features were selected by MRMD).

In this study, the Minimum Redundancy Maximum Relevance (mRMR)^30,31^, the analysis of variance (ANOVA) and Maximum Relevance Maximum Distance (MRMD)^32,33^ were applied on the expression profile of 117 immune-related genes, and the top 50 features, 84 features and 112 features were selected by these feature selection algorithms, respectively. These features were used as the input parameters of the naïve bayes, logistic, IBK, J48, random forest and libSVM. In the ten-fold cross validation, the overall accuracies of these algorithms with the default parameters in Weka were shown in Table 1. As shown in Table 1, the predictive results clearly indicated that these feature selection algorithms may improve the predictive results for IBK, J48 and libSVM.

In order to perform the cross platform data examination, the dataset of breast cancer was downloaded from TCGA, 1095 patients were contained in this cohort. Based on the expression levels of 117 immune-related genes, 2 clusters of TCGA breast cancer were identified by the Nbclust. Then, the expression profile of 117 immune-related genes was used as the input parameters of the shrunken centroid algorithm. In the ten-fold cross-validation, the overall accuracy of the shrunken centroid algorithm was 96.27%. The overall accuracies of the naïve bayes, logistic, IBK, J48, random forest, libSVM and shrunken centroid with the default parameter were 88.95%, 92.69%, 91.05%, 89.22%, 95.61%, 95.06% and 96.27%, respectively. Based on these results, we can conclude that our proposed model was suitable to predict the breast cancer patients in other platform data and its performance was better than other algorithms.

In this study, several limitations should be acknowledged. First, in our study, only the METABRIC breast cancer cohort was included in our analysis. Although, 1980 breast cancer patients were included in the METABRIC cohort, the dataset used here represented part but not all of the possible breast cancer presents. Since the TCGA breast cancer cohort and several GEO breast cancer cohorts were available on the website, more breast cancer cohorts were needed to confirm the effectiveness of our analysis. Second, the biological information on the mechanisms behind the immune-related genes was not clear, more experimental researches were needed to further understand their functional roles. Finally, there were more types of survival, such as progression-free survival, disease-free survival, and overall survival in the breast cancer cohorts, however, only the overall survival was used in this study. To yield more comprehensive analysis results for breast cancer patients, more types of survival should be used in our future work.

In summary, by employing the mRNA expression profile of the immune-related genes, our study demonstrated for the first time that the two molecular clusters of breast cancer patients. The approaches described here can conceivably be adapted for other tumors, and will provide a powerful tool for the systematic identification of immune-related biomarkers in clinical oncology. Prospective studies are needed to further validate our findings in prospectively planned clinical trials, and to test its clinical utility in individualized management of breast cancer.

## Material and Methods

### Breast cancer cohort

The relevant clinical data and gene expression data were retrieved from the METABRIC (Molecular Taxonomy of Breast Cancer International Consortium) breast cancer cohort^5^. Both the METABRIC training cohort and the METABRIC test cohort were used in this study. 1980 breast cancer patients with the clinical data, overall survival data and gene expression data were contained in our final cohort.

The mRNA expression data and clinical data of the breast cancer patients were downloaded from The METABRIC (Molecular Taxonomy of Breast Cancer International Consortium), which is public dataset for breast cancer patients, no experiments on humans and/or the use of human tissue samples were used in our study.

### Immune-related genes and immune infiltration signatures

2498 immune-related genes were downloaded from the ImmPort database^34^. 17 immune gene categories, such as antigen processing and presentation, antimicrobials, BCR signaling pathway, cytokine, interleukins, T-cell receptor signaling pathway, B-cell receptor signaling pathway and TNF family receptors were included in these immune-related genes. Subsequently, the single sample gene set enrichment analysis (ssGSEA)^35,36^ was used to calculate the abundance level of each gene category for each sample, and the normalized abundance level was considered as the tumor-infiltrating level (TIL) of each gene category for each sample.

The tumor purity, ESTIMATE score, immune score and stromal score were calculated by the ESTIMATE algorithm^8^. The CYT was calculated as the mean expression level of the granzyme A (GZMA) and perforin 1 (PRF1) for assessing the intratumoral immune cytolytic activity in tumors^10^.

### Breast cancer patients clustering

The subtypes of the breast cancer patients were identified by using the k-means clustering algorithm and the Nbclust that implemented in the R package factoextra (version 1.0.5) was used to determine the optimal number of stable breast cancer clusters. Silhouette width was computed to confirm the most stable samples within each cluster.

### Gene set enrichment analysis (GSEA)

To determine how the immune cell types differ between two breast cancer clusters in the tumor microenvironment, GSEA was performed by the R package clusterProfiler (version 3.4.1)^37^. All the immune-related genes that downloaded from the ImmPort database^34^ were selected as the reference gene set. Gene sets with the P-value less than 0.05 after 1000 permutations were considered to be significantly enriched or depleted. The normalized enrichment score (NES) was used to examine gene set enrichment results across different gene sets.

### Statistical analysis

Survival differences between two breast cancer clusters were assessed by the Kaplan-Meier estimate, and the differences between them were compared using the two-sided log-rank test. The univariable analysis and multivariate analysis were performed with the Cox proportional-hazards regression model. All statistical analyses were performed using R (version 3.6.1). All of the statistical tests were two-sided, and the significance was defined as P-values being less than 0.05.

## Supplementary information

Supplementary information.

## References

[1] Siegel RL, Miller KD, Jemal A (2018). Cancer statistics, 2018. CA Cancer J. Clin..

[2] Ali HR (2014). Genome-driven integrated classification of breast cancer validated in over 7,500 samples. Genome Biol..

[3] Ciriello G (2015). Comprehensive molecular portraits of invasive lobular breast cancer. Cell..

[4] Chuang HY, Lee E, Liu YT, Lee D, Ideker T (2007). Network-based classification of breast cancer metastasis. Mol. Syst. Biol..

[5] Curtis C (2012). The genomic and transcriptomic architecture of 2,000 breast tumours reveals novel subgroups. Nat..

[6] Prat A, Parker J, Fan C, Perou C (2012). PAM50 assay and the three-gene model for identifying the major and clinically relevant molecular subtypes of breast cancer. Breast cancer Res. Treat..

[7] Haibe-Kains B (2012). A three-gene model to robustly identify breast cancer molecular subtypes. J. Natl Cancer Inst..

[8] Yoshihara K (2013). Inferring tumour purity and stromal and immune cell admixture from expression data. Nat. Commun..

[9] Şenbabaoğlu Y (2016). Tumor immune microenvironment characterization in clear cell renal cell carcinoma identifies prognostic and immunotherapeutically relevant messenger RNA signatures. Genome Biol..

[10] Rooney Michael S, Shukla Sachet A, Wu Catherine J, Getz G, Hacohen N (2015). Molecular and genetic properties of tumors associated with local immune cytolytic activity. Cell..

[11] Ikeda Y (2017). Clinical significance of T cell clonality and expression levels of immune-related genes in endometrial cancer. Oncol. Rep..

[12] Yang L (2018). Clinical significance of the immune microenvironment in ovarian cancer patients. Mol. Omics.

[13] Stanton SE, Disis ML (2016). Clinical significance of tumor-infiltrating lymphocytes in breast cancer. J. Immunother. Cancer.

[14] Sato E (2005). Intraepithelial CD8+ tumor-infiltrating lymphocytes and a high CD8+/regulatory T cell ratio are associated with favorable prognosis in ovarian cancer. Proc. Natl Acad. Sci. USA.

[15] Gooden MJ, de Bock GH, Leffers N, Daemen T, Nijman HW (2011). The prognostic influence of tumour-infiltrating lymphocytes in cancer: a systematic review with meta-analysis. Br. J. Cancer.

[16] Tomšová M, Melichar B, Sedláková I, Šteiner I (2008). Prognostic significance of CD3+ tumor-infiltrating lymphocytes in ovarian carcinoma. Gynecol. Oncol..

[17] Nguyen N (2016). Tumor infiltrating lymphocytes and survival in patients with head and neck squamous cell carcinoma. Head. neck.

[18] Santoiemma PP, Powell DJ (2015). Tumor infiltrating lymphocytes in ovarian cancer. Cancer Biol. Ther..

[19] Deschoolmeester V (2010). Tumor infiltrating lymphocytes: an intriguing player in the survival of colorectal cancer patients. BMC Immunol..

[20] Dadmarz RD (1996). Tumor-infiltrating lymphocytes from human ovarian cancer patients recognize autologous tumor in an MHC class II-restricted fashion. Cancer J. Sci. Am..

[21] Ward M (2014). Tumour-infiltrating lymphocytes predict for outcome in HPV-positive oropharyngeal cancer. Br. J. Cancer.

[22] Rhee JK (2018). Impact of tumor purity on immune gene expression and clustering analyses across multiple cancer types. Cancer Immunol. Res..

[23] Tibshirani R, Hastie T, Narasimhan B, Chu G (2002). Diagnosis of multiple cancer types by shrunken centroids of gene expression. Proc. Natl Acad. Sci..

[24] Tomlins SA (2015). Characterization of 1577 primary prostate cancers reveals novel biological and clinicopathologic insights into molecular subtypes. Eur. Urol..

[25] Markert EK, Mizuno H, Vazquez A, Levine AJ (2011). Molecular classification of prostate cancer using curated expression signatures. Proc. Natl. Acad. Sci. USA.

[26] Golub T. R., Slonim D. K., Tamayo P., Huard C., Gaasenbeek M., Mesirov J. P., Coller H., Loh M. L., Downing J. R., Caligiuri M. A., Bloomfield C. D., Lander E. S. (1999). Molecular Classification of Cancer: Class Discovery and Class Prediction by Gene Expression Monitoring. Science.

[27] Sboner A (2010). Molecular sampling of prostate cancer: a dilemma for predicting disease progression. BMC Med. Genomics.

[28] Marisa L (2013). Gene Expression Classification of Colon Cancer into Molecular Subtypes: Characterization, Validation, and Prognostic Value. PLOS Med..

[29] Frank E, Hall M, Trigg L, Holmes G, Witten IH (2004). Data mining in bioinformatics using Weka. Bioinforma..

[30] Peng HC, Long FH, Ding C (2005). Feature selection based on mutual information criteria of max-dependency, max-relevance, and min-redundancy. IEEE Trans. Pattern Anal. Mach. Intell..

[31] Ding C, Peng HC (2005). Minimum redundancy feature selection from microarray gene expression data. J. Bioinf Comput. Biol..

[32] Zou Q, Zeng J, Cao L, Ji R (2016). A novel features ranking metric with application to scalable visual and bioinformatics data classification. Neurocomputing.

[33] Zou Q, Wan S, Ju Y, Tang J, Zeng X (2016). Pretata: predicting TATA binding proteins with novel features and dimensionality reduction strategy. BMC Syst. Biol..

[34] Bhattacharya S (2014). ImmPort: disseminating data to the public for the future of immunology. Immunologic Res..

[35] Barbie DA (2009). Systematic RNA interference reveals that oncogenic KRAS-driven cancers require TBK1. Nat..

[36] Hänzelmann S, Castelo R, Guinney J (2013). GSVA: gene set variation analysis for microarray and RNA-seq data. BMC Bioinforma..

[37] Yu GC, Wang LG, Han YY, He Q (2012). Y. clusterProfiler: an R package for comparing biological themes among gene clusters. Omics.

