# Supplementary material for: Molecular classification of breast cancer using the mRNA expression profiles of immune-related genes
Source: Sci Rep. 2020 Mar 16;10:4800. doi: 10.1038/s41598-020-61710-y (PMC7075995; doi:10.1038/s41598-020-61710-y)
Supplement: Supplementary file 1 — Supplementary information. [file 41598_2020_61710_MOESM1_ESM.docx]

**Molecular classification of breast cancer using the mRNA expression profiles of immune-related genes**

Juan Mei*, Ji Zhao, Yi Fu

School of Internet of Things Engineering, Wuxi City College of Vocational Technology, Wuxi, 214153 China

*To whom correspondence should be addressed

Juan Mei, Tel: +86 510 83276012; Fax: +86 510 83276012; Email address: meijuanwx@163.com

**
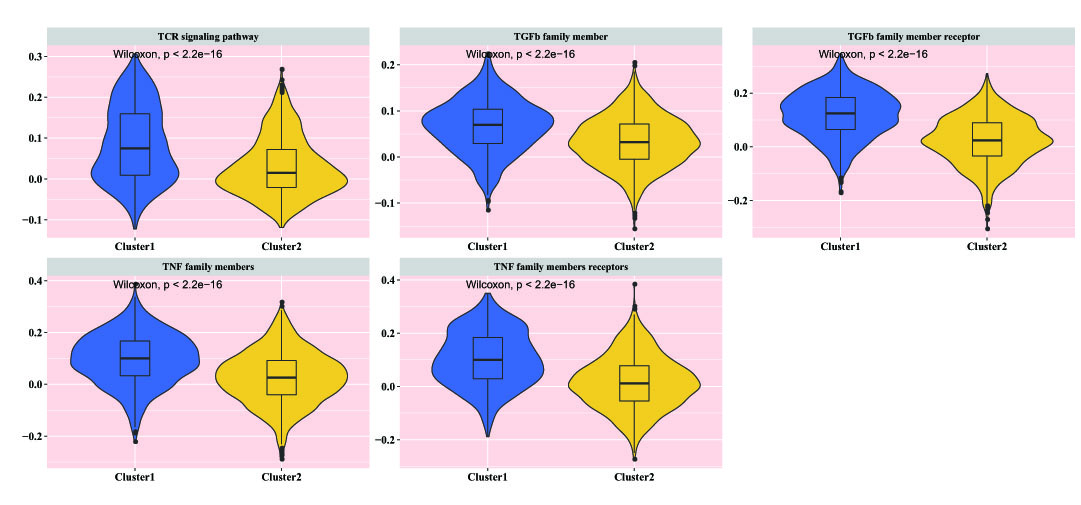
**

**Fig. S1** The violin plots of the tumor infiltrating levels in 5 immune cell types for in cluster 1 and cluster 2.


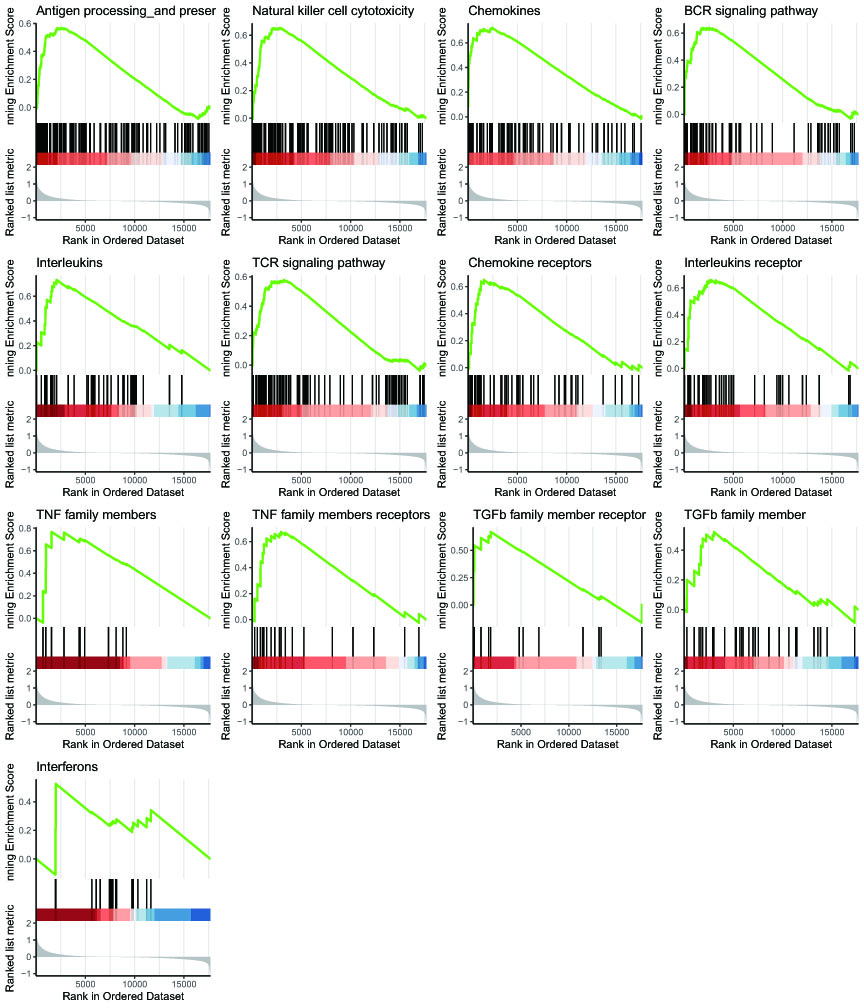


**Fig. S2** GSEA results for the cluster 1 when compared with the cluster 2.
